# Improved axenic hydroponic whole plant propagation for rapid production of roots as transformation target tissue

**DOI:** 10.1186/s13007-017-0189-z

**Published:** 2017-05-16

**Authors:** Kyle Benzle, Katrina Cornish

**Affiliations:** Department of Horticulture and Crop Science, The Ohio State University, 1680 Madison Avenue, Wooster, OH USA

## Abstract

**Background:**

Plant roots are used as an efficient target tissue for plant transformation assays. In root propagable species transformed roots are able to regenerate into whole plants without the addition of exogenous hormones, thus avoiding somaclonal variation associated with many plant transformation protocols. Plants grown in soil or soilless solid medium have roots that tend to be extremely delicate and are difficult to sterilize in advance of plant transformation experiments. Axenic tissue culture plants grown on semi-solid media are slow to produce large amounts of biomass compared to plants grown in solution-based media.

**Methods:**

Seeds were germinated and grown for 14 days on half-strength semi-solid Murashige and Skoog medium containing 1% sucrose. Seedlings were then transferred to Magenta™ GA7 vessels containing either liquid or semi-solid ½ MS medium with 0.25, 0.5, 1, 2 or 3% sucrose. In the hydroponics (liquid medium) treatments, expanded clay balls were used to anchor seedlings. Hydroponic vessels were fitted with a sterile air aeration hose and filled ¾ full (100 mL) with liquid ½ MS media. Liquid media were replaced after 7 days. All plants were grown under fluorescent lights for 14 days.

**Results:**

We have developed an improved axenic hydroponic propagation system for producing large quantities of plant roots for use in transformation assays using *Taraxacum kok*-*saghyz* as a model for root propagable species. Plants grew significantly faster in liquid media than on solid media. Addition of sucrose from 0.25 to 2% was correlated with an increase in biomass accumulation in plants grown in liquid media.

**Conclusions:**

Our improved axenic hydroponic method yields sufficient quantities of roots for extensive plant transformation/molecular studies.

## Background

*Taraxacum kok*-*saghyz* (TK) is a root propagable plant of interest because of the high quality natural rubber that is produced and stored in its roots [1, 2]. However, this species is recently wild-collected and modern molecular methods are needed to accelerate its domestication. Previously, a complimentary rapid plant transformation method was developed using *Agrobacterium rhizogenes* root transformation [3]. In both stable and transient root transformation methods, a common hurdle is the production of sufficient amounts of roots for experimentation. Traditionally, sterile tissue cultured plants are grown on semi-solid medium in autoclavable vessels which allow for microbe-free gas exchange through a vented lid. Plants grown under such conditions tend to grow slowly and methods must be optimized for each individual species, or even cultivar, targeted because species vary in their ability to regenerate under selection [4]. Cells targeted for transformation should be actively dividing and ideally near the tissue surface to be accessible to the transformation vector [5] and, in most cases, a high degree of replication is necessary to produce successful stable transformants.

Although hydroponic culture has been used successfully in many plants, overall adoption is low because the nutrient rich growth medium used encourages growth of unwanted micro-organisms and thus is difficult to maintain. Axenic plant hydroponic culture has generally been limited to cell culture or callus grown in suspension. However, axenic culture of whole plants has the added benefit of avoiding negative effects of somaclonal variation which can occur in cell and callus culture [6]. Whole plant cultures must be oxygenated but if this is accomplished using a shaker table, as is standard for callus tissue, delicate root structures may be damaged: such damage does not occur when a filtered aeration system is used [7]. Previously described systems have used glass jars and stainless mesh to anchor plantlets in liquid media [8] but these are difficult to maintain and do not produce large amounts of harvestable tissue. In this paper, we describe an easily implementable axenic culturing system. This system is suited to small-scale, rapid root production from seedlings to fully developed plants.

## Methods

### Growth media

In a preliminary experiment, TK tissue culture plants were grown on semi-solid media containing various concentrations of MS nutrients and sucrose. MS medium at 1× reduced adventitious root growth compared to 0.5 × MS medium. Therefore, in this study, solid and liquid media were made with 0.5 × Murashige and Skoog [9] medium (½ MS) (half strength MS micro- and macro-salts (Caisson Laboratories. North Logan, UT, USA) with 1 × Gamborg’s B5 vitamins and 10 g L^−1^ sucrose) [10] at pH 5.7 and 3.5 mM MES (25 mg L^−1^). Semi-solid growth media included plant tissue culture agar (Sigma-Aldrich, St. Louis, MO, USA) at 1% (w/v). Seeds, for both semi-solid and hydroponic cultures, were germinated on semi-solid ½ MS (see next section). Because 1% sucrose is the concentration normally used to screen genetically transformed plants [11], this was used to germinate seeds on semi-solid media (½ MS plates). Plants were grown in ½ MS with or without 1% agar at sucrose levels of 0.25, 0.5, 1, 2 and 3%.

### Plant material

All tissue manipulation was performed with ethanol-flamed forceps in a sterile laminar flow hood. *Taraxacum kok*-*saghz*, accession TK-17 [12] seeds were surface-sterilized in 25 mL 30% (v/v) domestic bleach with 0.03% Triton^®^ X-100 for 8 min at room temperature. The seeds were rinsed three times with 25 mL sterile double distilled water. About 100 seeds were sown on semi-solid ½ MS medium in Petri dishes (50 mL volume, 15 mm × 150 mm, Thermo Fisher Scientific Inc., Waltham, MA, USA) with 5–10 seeds per plate. Dishes were sealed with 3 M Micropore™ surgical tape (Thermo Fisher) and left for 48 h at 22 °C under ambient light. Seeded dishes then were placed under 16 h day fluorescent lighting conditions (PAR = 80–120 μmol m^−2^ s^−1^) for 7 days at 22 °C. Seedlings then were moved to either ½ MS liquid or semi-solid media in Magenta vessels and grown for an additional 14 days at 22 °C.

### Hydroponics apparatus

**Fig. 1:**
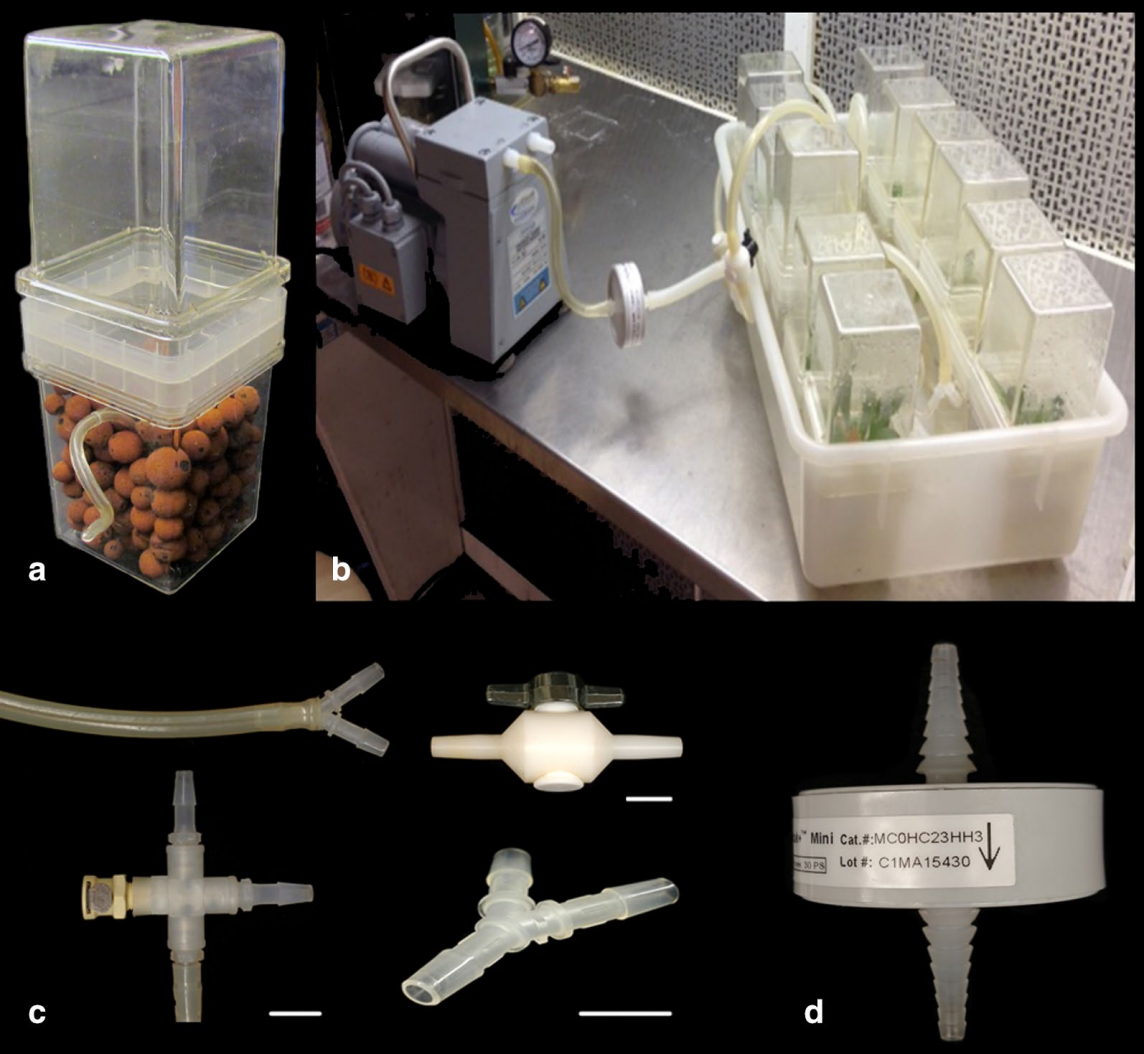
Hydroponics apparatus. Hydroponic apparatus consisting of: **a** liquid medium in autoclavable Magenta vessel with expanded clay balls fitted with 20 cm aeration hose. **b** A set of 10 vessels connected with Tygon tubing and aerated with air passed through a 20 μm filter. **c** Tygon tubing, connectors, splitters and valve used in the apparatus. **d** Millipore autoclavable filter. *Line* is approximately 1 cm

Modified Magenta G7 vessels were vertically connected using MK-5 10 mm Connector Lids (Caisson Laboratories) (Fig. 1a). The lower unit was filled with washed and autoclaved 10–30 mm expanded clay balls (Fig. 1b). A 5 mm hole was drilled in the top unit to insert a sterile air hose. Ten of the connected vessels were placed into a Nalgene™ Autoclavable Polypropylene Pan (Thermo Fisher) fitted with sufficient 5 mm Tygon tubing (Sigma-Aldrich) to reach the bottom of each vessel interior and have approximately 15 cm of tubing on the outside. Autoclavable Tygon splitters (Fig. 1c) were used to branch the single sterile air input into 10 separate lines, one for each vessel. The single air input line was fitted with an autoclavable 20 μM Acro^®^ 50 Vent Filter (Pall Laboratory, Port Washington, NY, USA) or Millipore Lab Millistak Mini, MCOHC23HH3, (Millipore Corporation, Billerica, MA) to sterilize incoming air (Fig. 1d).

Once fitted with the tubing, the entire apparatus was autoclaved and allowed to cool in a laminar flow hood before adding five treatment media in duplicate. Using aseptic technique, one seedling was placed in each of the 10 liquid medium treatment vessels and the 10 semi-solid medium filled vessels. The hydroponic apparatus was then fitted with a 12-W air pump and constantly aerated. The hydroponic vessels and semi-solid vessels were placed under 16 h day fluorescent lighting for 14 days at 22 °C. A range of sucrose concentrations was evaluated to maximize root growth. Media were replaced after the first 7 days, taking care to maintain sterile conditions. Tissue was harvested aseptically by sterilizing the outside of the vessel with 70% ethanol and removing the plants from their vessels using 30 cm forceps, briefly blotting them on sterile paper towels, and separating the roots and shoots using forceps. Root/shoot fresh weights and lengths were quantified using a balance (to nearest 0.01 g) and ruler (to nearest 1 mm).

## Results and discussion

**Fig. 2:**
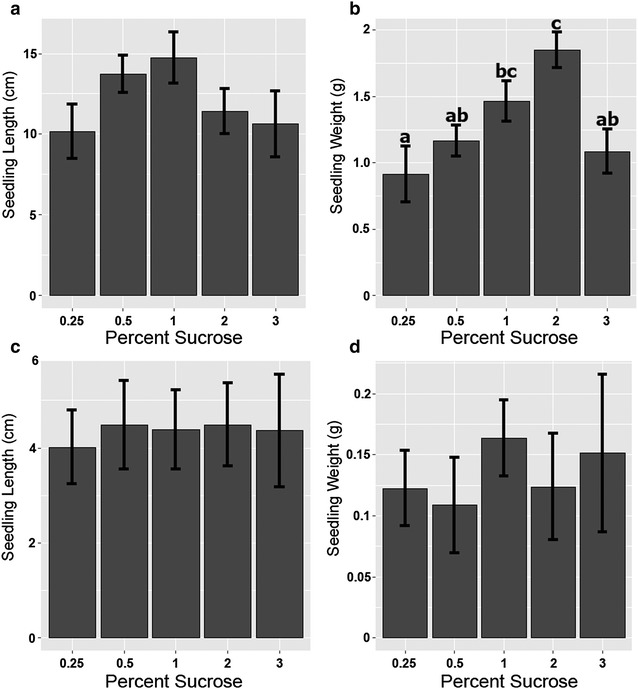
Comparison of hydroponic and semi-solid growth under varying sucrose levels. Analysis of root and shoot tissue under liquid (**a**, **b**) or semi-solid (**c**, **d**) culture treatments. **a** Seedling total length per individual plant for selected treatments. **b** Fresh weight biomass. **c** Seedling total length. **d** Fresh weight biomass. ANOVAs were conducted for all comparisons and Tukey’s HSD means comparison *letters* are shown where significant differences were found. *Letters* denote significant differences (*p* < 0.05), values are the mean ± SE (n = 8)

Hydroponically-grown plants grew up to 10 times larger than those on semi-solid media (Fig. 2a–d). Among treatments, the highest root and shoot biomass was achieved in hydroponically-grown TK plants in 2% sucrose with 0.5 × MS. One-way analysis of variance (ANOVA) was used to compare methods, Tukey’s Honestly Significant Difference (HSD) post hoc test was then used to compare groups and identify which samples differed significantly (with *p* < 0.05). Analysis was done with R v. 3.4.0 software (R Development Core Team, 2017). Statistically significant differences in plant root weight were found between sucrose levels of 0.25 and 3% when plants were grown in liquid culture that were not seen when a semi-solid medium was used.

**Fig. 3:**
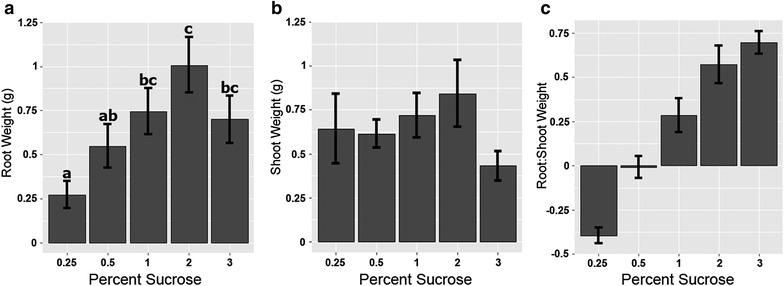
Hydroponic root-to-shoot weight ratios. Root and shoot fresh weights by percent sucrose in liquid media **a** root weight, **b** shoot weight, **c** root:shoot ratio. ANOVAs were completed for both comparisons and Tukey’s HSD means comparison *letters* are shown only where significant differences were found, values are the mean ± SE (n = 8)

**Fig. 4:**
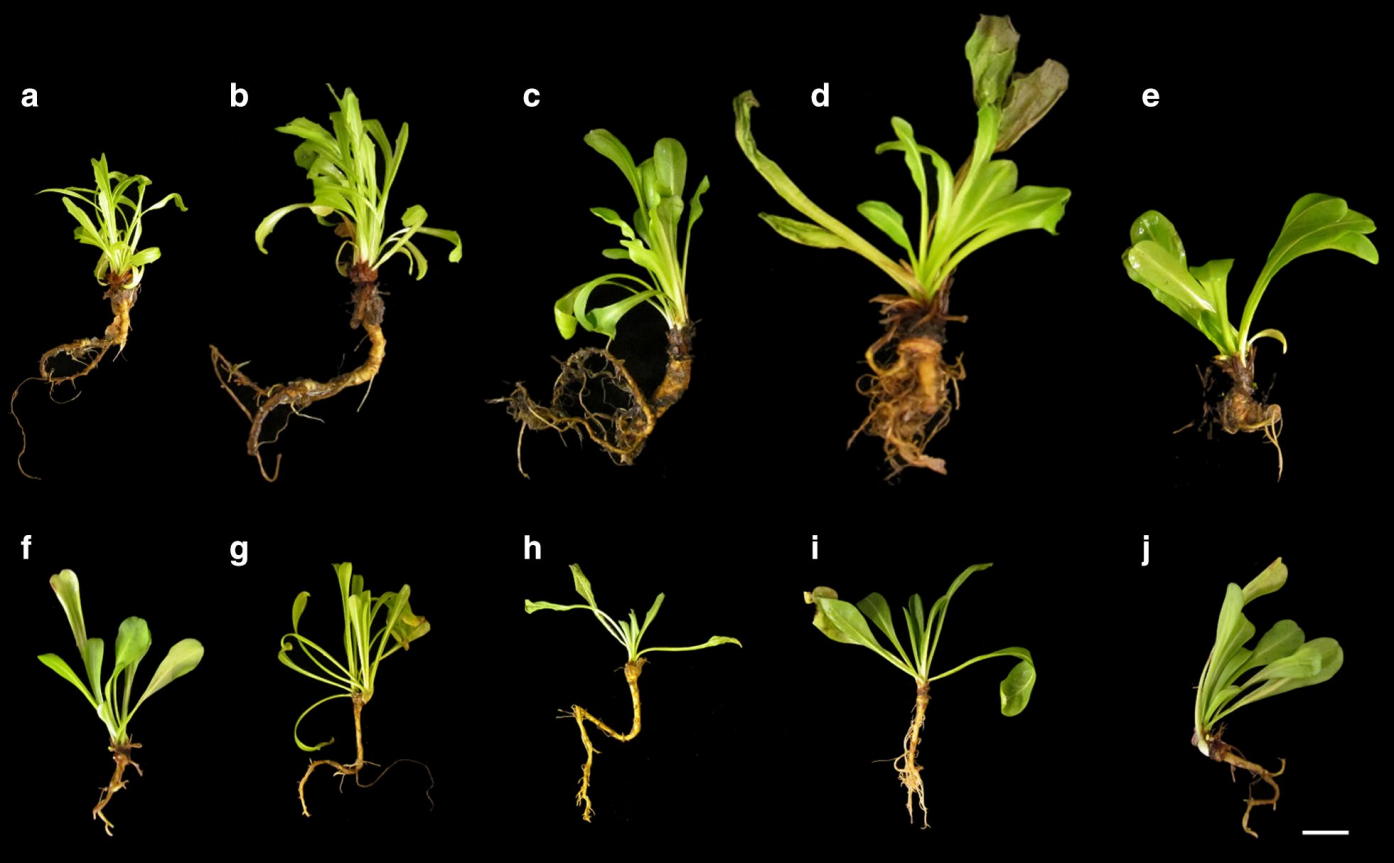
Hydroponic and semi-solid grown TK. TK seedlings, 21 days old, grown in liquid (**a**–**e**) and semi-solid (**f**–**j**) medium. *Letters* indicate sucrose levels of 0.25, 0.5, 1, 2 and 3% for **a**/**f**, **b**/**g**, **c**/**h**, **d**/**i** and **e**/**j** respectively. *Bar* 1 cm

All plants appeared to be morphologically normal, and primary and secondary roots were produced. In liquid medium, the largest plants were observed when plants were grown with 1–2% sucrose (Fig. 2a, b). However, the sucrose concentrations used in this experiment had very little effect on seedling growth on semi-solid media (Fig. 2c, d) although all supported more growth than no sucrose (data not shown). Growth of seedlings was inhibited by 3% sucrose in liquid media (Fig. 2b). Root biomass increased with the level of sucrose, but shoot biomass did not significantly increase (Fig. 3a–c). Visually, plants in liquid media (Fig. 4a–e) grew larger than those on semi-sold media (Fig. 4f–j), with biomass increasing more rapidly than seedling length, producing stouter plants. Nutrient levels and lighting conditions may be further optimized in future experiments.

## Conclusions

In conclusion, we have developed an improved and simple procedure for axenic plant culture. Statistically significant differences in plant root weight were found between sucrose levels of 0.25 and 3% when plants were grown in liquid culture that were not seen when a semi-solid medium was used. Expanded clay pebbles provide excellent anchorage for growing roots while also allowing easy harvest for subsequent subculturing or tissue analysis. Root biomass was increased by up to 10× by this liquid system compared to semisolid culture.

## References

[1] Schmidt T, Lenders M, Hillebrand A, van Deenen N, Munt O, Reichelt R (2010). Characterization of rubber particles and rubber chain elongation in *Taraxacum koksaghyz*. BMC Biochem.

[2] Cornish K (2001). Biochemistry of natural rubber, a vital raw material, emphasizing biosynthetic rate, molecular weight and compartmentalization, in evolutionarily divergent plant species. Nat Prod Rep.

[3] Zhang Y, Iaffaldano BJ, Xie W, Blakeslee JJ, Cornish K (2015). Rapid and hormone-free *Agrobacterium rhizogenes*-mediated transformation in rubber producing dandelions *Taraxacum kok*-*saghyz* and *T. brevicorniculatum*. Ind Crop Prod.

[4] Anami S, Njuguna E, Coussens G, Aesaert S, Van Lijsebettens M (2013). Higher plant transformation: principles and molecular tools. Int J Dev Biol.

[5] Birch RG (1997). Plant transformation: problems and strategies for practical application. Annu Rev Plant Biol.

[6] Karp A (1995). Somaclonal variation as a tool for crop improvement. Euphytica.

[7] Hetu M, Tremblay LJ, Lefebvre DD (2005). High root biomass production in anchored Arabidopsis plants grown in axenic sucrose supplemented liquid culture. Biotechniques.

[8] Alatorre-Cobos F, Calderón-Vázquez C, Ibarra-Laclette E, Yong-Villalobos L, Pérez-Torres C-A, Oropeza-Aburto A (2014). An improved, low-cost, hydroponic system for growing Arabidopsis and other plant species under aseptic conditions. BMC Plant Biol.

[9] Murashige T, Skoog F (1962). A revised medium for rapid growth and bio assays with tobacco tissue cultures. Physiol Plant.

[10] Gamborg OL, Miller R, Ojima K (1968). Nutrient requirements of suspension cultures of soybean root cells. Exp Cell Res.

[11] Bhojwani SS, Dantu PK (2013). Plant tissue culture: an introductory text.

[12] Hellier B (2011). Collecting in Central Asia and the Caucasus: US national plant germplasm system plant explorations. Hort Sci.

